# Autophagy, a Novel Pathway to Regulate Calcium Mobilization in T Lymphocytes

**DOI:** 10.3389/fimmu.2013.00179

**Published:** 2013-07-04

**Authors:** Wei Jia, Ming-Xiao He, Ian X. McLeod, You-Wen He

**Affiliations:** ^1^Department of Immunology, Duke University Medical CenterDurham, NC, USA

**Keywords:** autophagy, calcium flux, T lymphocytes, ER homeostasis, ER-phagy

## Abstract

The T lymphocyte response initiates with the recognition of MHC/peptides on antigen presenting cells by the T cell receptor (TCR). After the TCR engagement, the proximal signaling pathways are activated for downstream cellular events. Among these pathways, the calcium-signaling flux is activated through the depletion of endoplasmic reticulum (ER) calcium stores and plays pivotal roles in T cell proliferation, cell survival, and apoptosis. In studying the roles of macroautophagy (hereafter referred to as autophagy) in T cell function, we found that a pathway for intracellular degradation, autophagy, regulates calcium signaling by developmentally maintaining the homeostasis of the ER. Using mouse genetic models with specific deletion of autophagy-related genes in T lymphocytes, we found that the calcium influx is defective and the calcium efflux is increased in autophagy-deficient T cells. The abnormal calcium flux is related to the expansion of the ER and higher calcium stores in the ER. Because of this, treatment with the ER sarco/ER Ca^2+^-ATPase pump inhibitor, thapsigargin, rescues the calcium influx defect in autophagy-deficient T cells. Therefore, autophagy regulates calcium mobilization in T lymphocytes through ER homeostasis.

## Introduction

The highly conserved intracellular pathway, autophagy, degrades long-lived proteins, or damaged/extra organelles for quality control purposes to protects cells from death, or to provide energy during stress conditions (1). Using mouse genetic models, in which specific autophagy-related genes (Atgs) are deleted and autophagic pathways are blocked, our lab and other groups have found that autophagy-related molecules are expressed in T lymphocytes and T cell receptor TCR stimulation activates autophagy processing pathway (2–4). Autophagy developmentally regulates the homeostasis of endoplasmic reticulum (ER) and mitochondria (5, 6). ER is expanded when the autophagy pathway is impaired in T lymphocytes (7).

A physiological function of ER in T lymphocytes is the initiation of calcium flux after TCR engagement. The current model for calcium flux downstream of TCR activation is store-operated Ca^2+^ entry (SOCE) and this is mediated by the opening of Ca^2+^ release-activated Ca^2+^ (CRAC) channels on the T cell surface, which is in turn initiated by the depletion of ER calcium stores (8). Molecular mechanistic studies indicate that the ER-resident molecule, stromal interaction molecule 1 (STIM1), senses the calcium concentration of ER stores, redistributes itself and binds a pore subunit of CRAC, ORAI1, to begin the calcium influx into T cells (9–11). Calcium flux and signaling in T lymphocytes are tuned at different levels. We found that the calcium mobilization in T lymphocytes is also regulated by autophagy. Autophagy regulates the volume of the ER in both CD4^+^ and CD8^+^ T lymphocytes. Expanded ER leads to increased calcium stores when autophagy is impaired. Depletion of calcium stores is incomplete after TCR stimulation and the redistribution of STIM1 is severely reduced. Finally, calcium influx is much lower in autophagy-deficient T lymphocytes (7). Here we review how autophagy regulates the calcium mobilization in T lymphocytes.

## The Calcium-Signaling Pathway in T Lymphocytes

**Figure 1:**
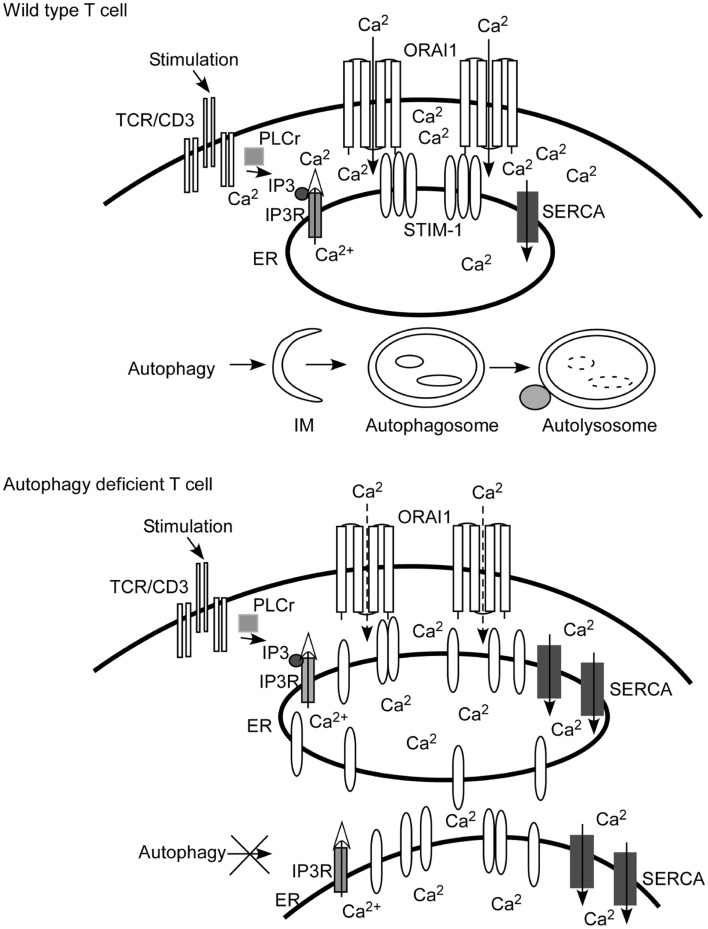
**Autophagy regulates calcium mobilization through the control of endoplasmic reticulum (ER) homeostasis**. Autophagy is activated when there are damaged, senescent, or extra organelles in order to maintain normal ER contents. In T lymphocytes, inositol 1,4,5-trisphosphate (IP3) is produced after TCR engagement. IP3 binds with the IP3 receptor (IP3R) expressed on ER to initiate the depletion of calcium stores from the ER lumen (marked with an open arrow head). The calcium sensor and ER-resident molecule stromal interaction molecule 1 (STIM1) oligomerizes, and redistributes toward the ER plasma membrane junction after the depletion of calcium stores. Then STIM1 interacts with the pore subunit of Ca^2+^ release-activated Ca^2+^ (CRAC) channels, ORAI1, to open CRAC channels. Extracellular calcium fluxes through CRAC channels into the cytoplasm of T cells (visualized as Ca^2+^ inside of the cells). When autophagy is ablated, as shown in the cartoon figure of autophagy-deficient T cells, the contents of the ER are expanded. Calcium stores are increased since the ER is expanded and more sarcoplasmic/endoplasmic-reticulum Ca^2+^-ATPase (SERCA) are expressed. The depletion of calcium stores is incomplete and less STIM1 redistributes to the ER plasma junction. Therefore, less CRAC is opened. The end result is that calcium influx is defective compared to that of wild type T cells. IM, isolation membrane.

After the initial TCR-MHC/peptide contact, activation of the Src-family tyrosine kinase, Lck, leads to the phosphorylation of tyrosine residues in the immunoreceptor tyrosine-based activation motifs (ITAMs) in CD3 chains of the TCR/CD3 complex. Following the phosphorylation of ITAMs, the Syk family kinase ZAP70 is recruited to the TCR/CD3 complex, phosphorylated, and activated by the tyrosine kinase, Lck. Next, ZAP70 phosphorylates and activates the linker for activation of T cells (LAT) and SLP-76. Then phosphatidylinositol-3-kinases (PI3K) are activated and phosphatidylinositol (3,4,5) triphosphate (PIP3) is produced. Following this, the inducible T cell kinase (Itk) is recruited and interacts with LAT and SLP-76 (12). This sequential cascade spreads, activating several different signaling pathways in the proximal signaling transduction in T lymphocytes. Among these pathways, calcium-signaling starts with the activation of phospholipase Cγ1 (PLCγ1) by Itk. PLCγ1 hydrolyzes phosphatidylinositol 4,5-bisphosphate (PIP2) to produce the secondary messengers inositol 1,4,5-trisphosphate (IP3) and diacylglycerol (DAG). DAG activates PKC-θ and MAPK/Erk pathways. IP3 binds to the IP3 receptor on the ER membrane to release calcium stores from the ER lumen (marked with open arrow head in Figure 1) in order to initiate calcium mobilization and activate further downstream signals in T lymphocytes (13).

The molecular mechanism of the opening of CRAC channels is mediated by the interaction between the ER-resident protein stromal interaction molecule (STIM) 1/2 (14) with CRAC channel components, ORAI protein (ORAI1 and its homologs ORAI2 and ORAI3) (10). ORAI1, ORAI2, and ORAI3 are widely transcribed in different tissues and ORAI1 is the dominant component of the CRAC channel in T lymphocytes (15, 16). Both STIM1 and STIM2 are expressed in T lymphocytes. However, STIM1 is the predominant regulator for SOCE in T cells, while the STIM2 plays a relatively less important role during SOCE (16, 17). STIM senses the concentration of ER calcium stores through an N-terminal EF-hand and a sterile α motif domain (EF-SAM) (18), forms oligomers, and redistributes itself toward a plasma membrane junction after calcium depletion from ER stores. Then the C-terminus of STIM1 interacts with the CRAC channel components ORAI1 to open the CRAC channels on the T cell surface (19). Consequent extracellular calcium influxes into T cells further activate downstream molecules of TCR signal transduction pathways. The influx of calcium activates the serine/threonine phosphatase calcineurin, which in turn phosphorylates nuclear factor of activated T cells (NFAT). NFAT translocates to nucleus to turn on the transcription of target genes, such as the cytokines IL-2, IL-17A, IL-22, IL-21, and the transcription factor Foxp3 depending on the situation (20–22). This signal transduction in T lymphocytes is finely regulated by different mechanisms.

## Autophagy in T Lymphocytes

Autophagy is a highly conserved cellular homeostasis and degradation pathway present in all eukaryotic species (23, 24). According to specific characteristics, three different types of autophagy have been described, termed microautophagy, macroautophagy, and chaperone-mediated autophagy (25). Most research focuses on macroautophagy. Macroautophagy degrades long-lived proteins, provides energy during stress conditions, maintains organellar homeostasis, and eliminates various invading intracellular pathogens (26). Panoply of cellular stress conditions, such as growth factor withdrawal, nutrient depletion, or T cell activation can activate the autophagy pathway. Autophagy starts with an overtly crescent membrane structure, called an isolation membrane (IM) in mammalian cells and a phagophore in yeast cells. These membranes are originally derived from Golgi membranes (27), plasma membrane (28), mitochondria (29), or ER (30).

In a manner remarkably homologous to the yeast system, two kinase complexes are essential for the induction of autophagy in mammalian cells. One is the class III PI3K complex and the other is UNC-51-like kinase (ULK) complex. The PI3K complex is composed of the class III PI3K catalytic subunit Vps34, the class III PI3-kinase regulatory subunit p150 (the homolog of Vps15 in yeast), Beclin 1 (the homolog of Vps30/Atg6 in yeast), and Barkor [Beclin 1-associated autophagy-related key regulator, also named KIAA0831 (31), or the Atg14-like molecule (Atg14L) (32), the homolog of Atg14 in yeast] (33). Several Beclin 1 interacted molecules, such as UV-irradiation-resistance-associated gene (UVRAG) (34), vacuole membrane protein 1 (VMP1) (35), activating molecule in Beclin 1-regulated autophagy 1 (Ambra1) (36), Bif-1 (37), and Rubicon (38) are also present in the PI3K complex and regulate autophagy. Vps34 phosphorylates phosphatidylinositol (PI) to produce phosphatidylinositol-3-phosphate (PI3P). The energy sensor, AMP-activated protein kinase (AMPK), phosphorylates T163/S165 of Vps34 to reduce the production of PI3P and therefore inhibits the induction of autophagy. While under conditions of nutrient stress, AMPK phosphorylates S91/S94 of Beclin 1 to activate the autophagic processing pathway. Atg14L distinguishes between nutrient rich or starvation conditions through the inhibition of the phosphorylation of Vps34 induced by AMPK, but promotes the phosphorylation of Beclin 1 caused by AMPK under starvation conditions (39). In T lymphocytes, Vps34 controls the trafficking, recycling, and signaling capacity of the IL-7 receptor (IL-7R), which provides a major survival signal for naïve T cells (40). In another model, Vps34-deficient T cells showed impaired autophagy and abnormal homeostasis of mitochondria (41). The ULK complex includes the mammalian Atg13, FIP200 (Atg17 in yeast) (42), Atg101 (43, 44), and one ULK1 or one ULK2. ULK is the homologous molecule of the serine/threonine kinase Atg1 in yeast. mTOR phosphorylates Atg13, ULK1, and ULK2, and inhibits ULK1 and ULK2 kinase activity to inhibit autophagy induction. Atg13 mediates the interactions between ULK1/2 and FIP200, and is essential for the phosphorylation of FIP200 by ULK (45). Atg101 is required for the stability and phosphorylation of ULK and Atg13 (43, 44).

During the elongation phase, the IM is further expanded and directed by autophagy-related molecules to form a characteristic double membrane structure, termed an autophagosome, to enwrap cytosolic materials. The enveloped components of the autophagosome can be long-lived proteins, organelles, or even invading pathogens (46). Two protein/lipid conjugation systems mediated by Atg molecules regulate autophagosome formation outward from the IM structures. One is the Atg12-conjugation system and the other is the microtubule-associated protein 1 light chain 3 (LC3, Atg8 in the yeast system)-conjugation system. Atg7, an ubiquitin E1-like molecule, is involved in both conjugation systems. Atg10 and Atg3 are ubiquitin E2-like molecules and participate in either the Atg12 or LC3-conjugation system, respectively. Atg12, Atg5, and Atg16L form a large complex (47), the culmination of the Atg12-conjugation system, which further functions as an ubiquitin E3-like molecule to enhance the formation of the lipid form of phosphatidylethanolamine (PE)-LC3 (LC3-II) in the LC3-conjugation system. The lipid form of LC3 (LC3-II) is widely used as a marker for the detection of autophagy induction (48). Finally, at the maturation stage, the autophagosomes fuse with preexisting lysosomes to become mature autolysosomes and lysosomal enzymes degrade the enclosed materials. Macromolecular transporters in the autolysosome then allow for the recycling of degraded materials back to the cytoplasm (49, 50).

The discovery of autophagy related to the adaptive immune system was first reported in the late 1960s. Abnormal granules were observed in human lymphocytes from sarcoidosis patients treated with chloroquine and these granules in the cytosol of lymphocytes were hypothesized as autophagy-related structures (51). In 1984, Seglen first identified that there was autophagosome formation in human primary lymphocytes as well as in leukemic cells (52). In 2004, Gerland reported that several Atgs were expressed in long-term (>14 weeks) cultured human CD8^+^ T cells. Autophagy was induced in these senescent cells and related to cell death (53). In 2006, Espert found that HIV-1 envelope glycoproteins induced autophagy and accumulation of Beclin 1 in HIV-uninfected CD4^+^ T cells through CXCR4 to cause cell death (54). Our lab and other groups have thoroughly analyzed the autophagic processing pathways in mouse T lymphocytes using mouse genetic models (2, 3, 55). Many Atgs, such as Atg5, Beclin 1 and LC3, are expressed in thymocytes, most highly during double negative (DN) thymocyte development, but also expressed in mature CD4^+^ and CD8^+^ T cell sub-populations. Both CD4^+^ and CD8^+^ lymphocytes continue expressing autophagy genes after TCR stimulation and activation. The expression of autophagy machinery was further confirmed by the observation of characteristic double membrane structures of autophagosomes in T lymphocytes by electron microscopy (EM). Compared to freshly isolated T lymphocytes, the formation of LC3-II was moderately promoted by starvation, but strongly induced by anti-CD3 antibody-mediated TCR stimulation. The detection of LC3-II indicates that autophagic flux occurs in T lymphocytes after T cells are activated by TCR stimulation (3). By using mouse genetic models in which Atg5 (3, 6), Atg7 (5, 55), Atg3 (56), Vps34 (41), or Beclin 1 (57), were specifically deleted in T lymphocytes, it is apparent that autophagy developmentally regulates the homeostasis of organelles such as mitochondria or ER in T lymphocytes (5–7). Through the use of BAC Beclin 1-GFP transgenic mice, Arsov reported that the expression of Beclin 1 was developmentally regulated in both T and B lymphocytes. Beclin 1-GFP is highly expressed in DN thymocytes, down-regulated in double positive (DP) thymocytes and re-expressed in mature thymocytes (4). On top of this, recombination activating gene 1 (Rag1)^−*/*−^ chimeric mice reconstituted with Beclin 1^−*/*−^ embryonic stem cells (ESCs) indicated that Beclin 1 is involved in the development of early progenitors of thymocytes (58). The functions of autophagy in T lymphocytes have been reviewed in detail (59, 60). Autophagy is essential for the survival of mature T lymphocytes (3, 56). More specifically, autophagy regulates calcium mobilization in T lymphocytes (7).

## Autophagy Developmentally Regulates the Homeostasis of ER in T Cells

One of the basic physiological functions of autophagy is to remove damaged, senescent, or extra organelles before they become cytotoxic. Contrary to the non-selective bulk degradation of cytosol materials, autophagy selectively reduces organelles to maintain homeostatic volumes. Selective autophagy for the degradation of ER and mitochondria are termed as ER-phagy (or reticulophagy) (61, 62) and mitophagy (63), respectively. ER-phagy can be induced by starvation or the unfolded protein response (UPR). ER-phagy eliminates the expanded ER volume when the UPR is not needed (62). Our data suggests that autophagy maintains the volumes of organelles in certain levels at different stages during T cell development. Analysis of mouse genetic models demonstrates that the deficiency of Atg5, Atg7, Atg3, or Vps34 blocks the autophagy machinery in T lymphocytes. Both the mitochondrial contents and ER volumes are abnormal in autophagy-deficient thymocytes and mature CD4^+^ and CD8^+^ T cells (5–7, 41, 56).

During thymocyte development, the contents of ER are dynamic. The thymocytes at DN stage have highest level of the ER volumes and ER content decreases at the DP and single positive (SP) stages. Mature T cells have relatively lower ER contents. Autophagy-deficient thymocytes have similar ER contents in the DN, DP, and SP thymocytes compared to that of wild type thymocytes. However, the ER contents expand in both mature CD4^+^ and CD8^+^ autophagy-deficient T cells. Therefore, autophagy maintains ER membrane and content at relative lower levels in mature T cell populations (7). In an inducible-deletion system, the level of ER or mitochondria membranes start increasing at day 10 and significantly increase by day 21 after Atg3 is inducibly deleted and autophagy processing pathway is blocked. Therefore, autophagy regulates the homeostasis of the ER in a temporal manner (56).

Autophagy provides protective roles for cell survival during ER stress (64). Autophagy-deficient T cells constitutively express ER-stress markers, such as disulfide isomerase (PDI), and ER chaperones, such as glucose-regulated protein 78 (Grp78), and Grp94 (7). This suggests that the ER-stress response is activated in autophagy-deficient T cells and ER-stress caused by abnormal homeostasis of ER is one of the reasons why autophagy-deficient T cells show increased susceptibility to apoptosis.

## Calcium Stores are Increased in Autophagy-Deficient T Cells

One of the main functions of the ER in T lymphocytes is to regulate calcium mobilization. Upon TCR engagement, the calcium flux starts with the depletion of calcium stores in the ER lumen. The calcium stores are dramatically increased in autophagy impaired T lymphocytes. The higher calcium stores in ER are consistent with the expansion of ER contents in autophagy-deficient mature CD4^+^ and CD8^+^ T cells. The calcium stores are maintained by the sarcoplasmic/endoplasmic-reticulum Ca^2+^-ATPase (SERCA) pumps expressed on the surface of the ER. Autophagy-deficient T cells express twofold more SERCA pumps than wild type T cells (7). Over the life span of the cell, more calcium is imported by the SERCA pumps in autophagy-deficient T lymphocytes, which leads to increased calcium stores. Higher expressed SERCA pumps also affect the depletion of calcium stores in ER after TCR engagement in autophagy-deficient T cells.

The abnormal and excessive calcium stores in the ER and defective depletion directly affect the oligomerization and redistribution of the calcium sensor STIM1. Although autophagy-deficient T cells express more STIM1, the puncta intensity of STIM1 after TCR stimulation is much lower in autophagy-deficient T cells than that of wild type cells. The autophagy-deficient T cells express a similar level of ORAI1 compared to that of wild type T cells and CRAC channels remain intact in autophagy-deficient T cells (7). Therefore, the insufficient opening of CRAC channels is caused by the higher calcium stores, incomplete depletion, and less oligomerization of STIM1 after TCR activation in autophagy-deficient T cells.

## Autophagy Regulates the Calcium Mobilization through the Control of ER Homeostasis in T Cells

Mouse genetic models provide novel methods to investigate the physiological functions of autophagy. The specific deletion of Atg7, Atg3, or other Atgs blocks the autophagy processing pathway. The calcium influx in autophagy-deficient T cells is defective upon receipt of TCR signaling. The reason behind the calcium influx defect is due to the higher calcium stores mediated by the expansion of ER organelles in autophagy impaired T lymphocytes. The SERCA pump inhibitor, thapsigargin, inhibits the SERCA pumps from taking up calcium, corrects oligomerization of STIM1, and rescues the defective calcium influx in autophagy-deficient T lymphocytes. A model of the regulation of calcium mobilization in T lymphocytes by autophagy is summarized in Figure 1. Individual cell calcium influx analysis indicates that it takes longer for calcium influx in autophagy-deficient T cells to reach the peak of [Ca^2+^]_i_, in addition to less total calcium influx after stimulation. The average time for wild type cells to reach the peak of [Ca^2+^]_i_ is 56 s, while it takes 76 s for autophagy-deficient T cell to reach a lower peak of [Ca^2+^]_i_ (7).

Autophagy also regulates the homeostasis of mitochondria (5, 6). In contrast to the constant trimming of the ER, autophagy decreases the contents of mitochondria from SP compartment of thymocytes to mature CD4^+^ or CD8^+^ T cells in a developmentally stage-specific manner. Although there is abnormal expansion of total mitochondrial levels in autophagy-deficient T cells and mitochondria also contributes to the regulation of calcium flux through taking up calcium from the cytosol, the defect of calcium influx in autophagy-deficient T cells is not related to the abnormal expansion of mitochondria. When autophagy-deficient T cells were treated with carbonyl cyanide *m*-chlorophenylhydrazone (CCCP) before or after stimulation with thapsigargin, the calcium influx was not different between wild type and autophagy-deficient T cells (our unpublished data).

Although the calcium storage and influx is defective in autophagy-deficient T cells, the IL-2 production is not decreased and actually more IL-2 is produced in autophagy-deficient T cells. Since the calcium influx is not totally abolished in autophagy-deficient T cells, the observed level of calcium proves to be sufficient for turning on the transcription and translation of IL-2 (7). However, Hubbard reported that upon activation CD4^+^ T cells, IL-2, and IFN-γ production were defective in Atg7-deficient T cells (55). A recent study demonstrates that the autophagy adaptor protein, p62, is important for the ability of Bcl10 to signal to NF-κB, but also for its degradation by autophagy, explaining the enhanced IL-2 production by autophagy-deficient T cells (65).

The regulation of the ER by autophagy is not completely surprising. The ER is one of the purported sites of membrane donor activity for autophagy (66). Additionally, ER stress and the UPR are potent inducers of autophagy (67, 68). The knockdown of inositol trisphosphate receptor (IP3R) expressed on ER membranes or treatment with an IP3R antagonist can induce autophagy (69). Therefore, when autophagy is genetically inhibited for long periods of time, autophagosomes are not formed from the ER-mitochondrial membrane junctions. Since the ER membranes are not trimmed to provide substrates for the elongation of autophagic membranes, they accumulate and express ER-stress markers (7). This process is especially important in cells that make large amounts of secreted proteins, such as plasma cells (70, 71), but also in cells with very little cytoplasmic volume, such as naïve T cells (7). Thus, autophagy is a pro-survival stress response.

## Autophagy-Related Calcium Homeostasis is Involved in the Pathogenesis of Diseases

A mutation in the gene encoding α-synuclein has been shown to be related to the familial forms of Parkinson disease. Cellular α-synuclein maintains the morphology of mitochondria and regulates pools of Ca^2+^ transferred from ER stores to the mitochondria. Homeostatic levels of α-synuclein control the uptake of calcium by mitochondria. Autophagic flux is enhanced when calcium uptake in mitochondria is reduced, due to the inability of mitochondria to buffer Ca^2+^ concentrations (72). Another report indicates that increased intra-axonal calcium levels are followed by the activation of autophagy-mediated axonal degeneration, which often accompanies traumatic nerve injury or neurodegenerative diseases (73). Autophagy induced by calcium signaling is also involved in cell survival during hypoxia-induced stress. In a mouse liver ischemia-reperfusion injury model, the Ca^2+^/calmodulin-dependent protein kinase IV (CaMKIV) is activated and induces autophagy to protect hepatocytes from oxidative-stress-induced cell death (74).

In cancer cells, autophagy is often associated with enhanced cell survival. In breast cancer cells, nutrient and growth factor withdrawal decreases ATP and activates Ca^2+^/calmodulin-dependent protein kinase III, the eukaryotic elongation factor-2 kinase (eEF-2 kinase). Finally, autophagy provides protective roles for cancer cells. Knockdown of eEF-2 kinase inhibits autophagy and imparts sensitivity of breast cancer cells to treatments based on the inhibition of growth factors (75). However, the plant indole, diindolylmethane, found in cruciferous vegetables, has antineoplastic activity through the regulation of autophagy to attenuate the growth of cancer cells. Diindolylmethane induces ER stress in ovarian cancer cells and increases cytosolic calcium, which activates AMPK. The activation of AMPK promotes autophagy and inhibits ovarian cancer cell growth (76). Thus autophagy helps cells adapt to ever changing cellular conditions related to stress, metabolism, but acts as a brake on uncontrolled proliferation.

## Conclusion

Autophagy regulates the homeostasis of ER in a temporal manner. Abnormal expansion of ER increases the calcium stores in the ER lumen. The excessive calcium stores cause the incomplete depletion of resident ER calcium stores and directly affect the oligomerization and redistribution of STIM1 upon TCR activation. Finally, CRAC channels cannot be opened completely and eventually the calcium influx is much lower after T cells are activated. This suggests that autophagy is a novel pathway to regulate the calcium mobilization in T lymphocytes. When published data are taken into consideration, it is apparent that increased cytosolic calcium could inhibit mTOR to induce autophagy in human tumor cell lines and this pathway is mediated by Ca^2+^/calmodulin-dependent kinase kinase-β (CaMKK-β) and AMPK. Ectopic expression of Bcl-2 in ER decreased the calcium stored in the ER and inhibited the autophagy induced by increased cytosolic calcium (77). The inhibition of the calcium-signaling impacts autophagy. In T cell lines, it has been demonstrated that glucocorticoids promote autophagy through the downregulation of Fyn and inhibition of IP3-mediated calcium signaling (78). It seems that the autophagic pathway and calcium mobilization are reciprocal and delicately intertwined.

## Conflict of Interest Statement

The authors declare that the research was conducted in the absence of any commercial or financial relationships that could be construed as a potential conflict of interest.
